# Use of Information and Communication Technologies in Patients With Cancer Receiving Antineoplastic or Supportive Therapy: Comparative Cross-Sectional Survey

**DOI:** 10.2196/90339

**Published:** 2026-08-06

**Authors:** Roberto Collado-Borrell, Vicente Escudero-Vilaplana, Antonio Prieto-Romero, Andres Jesus Muñoz Martin, Marta Cantero-Martín, Cristina Villanueva-Bueno, Jose Luis Revuelta-Herrero, Ana Herranz-Alonso, Maria Sanjurjo-Saez

**Affiliations:** 1 Hospital General Universitario Gregorio Marañón, Instituto de Investigación Sanitaria Gregorio Marañón, Doctor Esquerdo, 46, Madrid, 28007, Spain, 34 915867714

**Keywords:** mobile health, digital health, oncology, mobile apps, telemedicine, digital literacy, patients with cancer, health communication, survey

## Abstract

**Abstract:**

**Background:**

The expansion of information and communication technologies (ICTs) has transformed the way patients access health information and manage their disease. In oncology and hematology care, the use of digital tools and mobile health has increased, especially after the COVID-19 pandemic, although challenges remain regarding adoption, trust, and perceived utility.

**Objective:**

This study aimed to evaluate the current use of ICTs by patients with cancer receiving antineoplastic and/or supportive therapy, to assess their interest in mobile apps for disease management, and to compare these findings with those obtained in a previous survey conducted in 2017.

**Methods:**

A comparative cross-sectional survey was conducted in a 1300-bed university hospital between August and December 2023. Adult patients with solid tumors or hematological malignancies receiving antineoplastic and/or supportive therapy were invited to complete a structured paper questionnaire based on a previous survey conducted in 2017. The questionnaire included 30 items regarding sociodemographic and clinical characteristics, ICT use, preferences concerning health apps, and the impact of the COVID-19 pandemic. Descriptive and inferential analyses were performed to compare results between the prepandemic survey conducted in 2017 (2017 study group; n=612) and the postpandemic survey conducted in 2023 (2023 study group; n=690). Missing responses were reported as “no response” and were not imputed.

**Results:**

Smartphone use increased from 82.5% (505/612) to 91% (628/690; *P*<.001), and the proportion of patients with installed health apps rose from 20.3% (124/612) to 53.9% (372/690; *P*<.001). Familiarity with technological terms and the use of connected devices also improved. However, full trust in online health information remained low (64/690, 9.3%), with most patients reporting trust contingent on source credibility. Preference for videoconferencing as a communication channel with health care professionals increased from 19% (116/612) to 32% (221/690; *P*<.001), while the use of email and mobile apps for this purpose declined. Willingness to use apps recommended by health care professionals remained high, although it decreased slightly from 81.4% (498/612) to 77.8% (537/690).

**Conclusions:**

The use of ICTs and digital health tools among patients with cancer has increased significantly in recent years, particularly after the COVID-19 pandemic. Nevertheless, barriers persist in terms of source credibility, usability, digital skills, and alignment with patient preferences. Strategies such as clinician-curated information sources, co-designed apps, simple communication channels, and targeted digital literacy support may help integrate these technologies more effectively and equitably in cancer care.

## Introduction

### Background

The expansion of information and communication technologies (ICTs) has significantly transformed the way the population accesses health information. In Spain, more than 66% of the general population possesses advanced digital skills, and the use of mobile apps is widely extended [1]. However, this estimate may not fully reflect the digital profile of patients with cancer, who are often older and may have heterogeneous levels of digital literacy and support needs. Worldwide, it is estimated that there are 337,000 health-related apps, many of which are designed for symptom monitoring, access to medical information, or communication with health care professionals [2]. This development is particularly relevant in the context of population aging and the increasing prevalence of chronic diseases, such as cancer. Patients with cancer require more personalized care, effective communication channels, and access to remote health care services. In this scenario, mobile health (mHealth), as a component of ICTs, has emerged as a key tool by enabling the collection, processing, and transmission of health information through apps and telemonitoring devices [3-5].

In a survey conducted in 2017 with more than 600 patients with solid tumors or hematological malignancies receiving antineoplastic treatment, 87.1% reported being interested in staying informed about health-related issues, and 61.3% actively sought health information online. Although 82.7% owned a smartphone, only 20.3% had installed a health-related app [6].

The COVID-19 pandemic represented a turning point in the adoption of digital health technologies. During the early stages, weekly telemedicine visits through the Medicare program in the United States increased by 153% [7]. Other studies have also described a rapid expansion of telehealth during the COVID-19 pandemic, suggesting a relevant and sustained role for these technologies in future health care delivery [8]. This digital transformation has greatly facilitated interactions between patients and health care professionals, consolidating telemedicine as a real and sustainable model of care [9].

Nevertheless, barriers remain that hinder its widespread implementation. A total of 62.5% of users consider that digital health services greatly depersonalize medical care. Furthermore, the lack of digital skills represents a significant limitation, with 75.7% of users acknowledging that these tools are not easily accessible for people with limited digital literacy [10]. In this context, the existence of clear evidence and regulatory frameworks is directly related to patient trust, source credibility, and the role of health care professionals when recommending digital tools [11].

### Objectives

We aimed to evaluate the current use of ICTs by patients with cancer receiving antineoplastic and/or supportive therapy, to assess their interest in apps for disease management, and to examine the temporal evolution of these aspects by comparing the 2023 survey with the previous survey conducted in 2017.

## Methods

### Study Design

A comparative cross-sectional survey was conducted to evaluate the use of ICTs among patients with cancer and its temporal evolution compared with a previous survey conducted in 2017 [6]. The 2023 survey was administered to adult patients with solid tumors or hematological malignancies receiving antineoplastic therapy (chemotherapy, immunotherapy, oral targeted therapies, or hormone therapy) and/or supportive care at a 1300-bed university hospital. Patients were approached during routine care in the day hospital or outpatient hospital pharmacy consultations. Patients aged <18 years or those unable to understand the questionnaire due to language or cultural barriers were excluded. Because participation depended on attendance at these care areas and willingness to complete a paper questionnaire, selection bias cannot be ruled out.

### Questionnaire

The survey was conducted between August and December 2023. A paper copy of the questionnaire was distributed to eligible patients in the day hospital or during outpatient hospital pharmacy consultations. Patients could complete the questionnaire onsite or at home and return it at their next visit.

The questionnaire was designed by a multidisciplinary team including 3 pharmacists and 2 oncologists with expertise in the application of ICTs in oncology (Multimedia Appendix 1). It was based on a previous survey conducted in 2017 by Collado et al [6], which was used as the comparative core questionnaire. The 2017 items were maintained to allow comparison between periods, and 3 new questions were added to assess the impact of the COVID-19 pandemic on ICT use among patients with cancer. The final questionnaire consisted of 30 items organized into four sections: (1) patient sociodemographic and clinical characteristics (items 1‐8), (2) use of ICTs to search for health information (items 9‐21), (3) preferences regarding the use of health apps (items 22‐27), and (4) impact of COVID-19 on ICT use (items 28‐30).

The questionnaire included dichotomous variables (items 2, 3, 6, 12, 18, 20, and 22) with true or false response options, as well as categorical polytomous variables (items 4‐5, 7‐10, 11, 13‐17, 19, 21, and 23‐30). To assess comprehension, 4 patients completed a draft version of the questionnaire. This pilot assessment was intended to identify comprehension problems and was not a formal psychometric validation.

### Ethical Considerations

The study protocol was approved by the hospital’s research ethics committee (CHPHDO2022) and was conducted in accordance with the ethical principles of the Declaration of Helsinki. No personal patient data were collected to ensure confidentiality.

### Statistical Analysis

Descriptive statistics were used to summarize the study population. Categorical variables were expressed as frequencies and percentages. Percentages were calculated using the total number of participants in each period, and missing answers were reported as “no response” in the tables. No imputation of missing responses was performed. To compare the distribution of variables between the 2 study periods (prepandemic survey, 2017 study group vs postpandemic survey, 2023 study group), Pearson chi-square test was used. In cases where the expected frequency in any cell of the contingency table was <5, Fisher exact test was applied. All statistical tests were 2-tailed, and a *P* value of <.05 was considered statistically significant. All data analyses were performed using the R statistical software environment (R Foundation for Statistical Computing).

## Results

### Participant Characteristics

A total of 711 questionnaires were distributed to patients with cancer, with a response rate of 97% (690 questionnaires were evaluated). The results were compared with the 612 questionnaires collected in the prepandemic survey (2017 study group) study. The sociodemographic and clinical characteristics of the patients in both periods are summarized in Table 1.

**Table 1.** Sociodemographic characteristics of patients.

Characteristics	Prepandemic survey (2017 study group; n=612), n (%)	Postpandemic survey (2023 study group; n=690), n (%)	*P* value
Sex			.02
Male	233 (38.1)	306 (44.3)	
Female	378 (61.8)	382 (55.4)	
No response	1 (0.2)	2 (0.3)	
Age (years)			.09
≤55	243 (39.7)	225 (32.6)	
56-65	179 (29.2)	210 (30.4)	
>65	176 (28.8)	214 (31)	
No response	14 (2.3)	41 (5.9)	
Lives alone			.001
Yes	61 (10)	112 (16.2)	
No	547 (89.4)	571 (82.8)	
No response	4 (0.7)	7 (1)	
Uses a tele-homecare system^a^			.99
No	576 (94.1)	647 (93.8)	
Yes	35 (5.7)	40 (5.8)	
No response	1 (0.2)	3 (0.4)	
Level of education			.29
No education or incomplete primary education	32 (5.2)	22 (3.2)	
Primary education	110 (18)	128 (18.6)	
Secondary education	220 (35.9)	262 (38)	
University education	249 (40.7)	276 (40)	
No response	1 (0.2)	2 (0.3)	
Some degree of dependence			.73
No	528 (86.3)	599 (86.8)	
Needs help once a day	29 (4.7)	33 (4.8)	
Needs help several times a day	40 (6.5)	46 (6.7)	
Totally dependent	13 (2.1)	9 (1.3)	
No response	2 (0.3)	3 (0.4)	
Type of cancer			
Bladder	9 (1.5)	19 (2.8)	.16
Breast	124 (20.3)	129 (18.7)	.52
Cervical	8 (1.3)	5 (0.7)	.44
Colorectal	113 (18.5)	108 (15.7)	.20
Esophageal	13 (2.1)	9 (1.3)	.35
Head and neck	16 (2.6)	20 (2.9)	.89
Hematological	3 (0.5)	54 (7.8)	<.001
Kidney	16 (2.6)	28 (4.1)	.20
Lung	60 (9.8)	80 (11.6)	.34
Ovarian	31 (5.1)	39 (5.7)	.73
Pancreatic	28 (4.6)	43 (6.2)	.23
Prostate	12 (2.0)	51 (7.4)	<.001
Sarcoma	16 (2.6)	38 (5.5)	.01
Skin or melanoma	8 (1.3)	9 (1.3)	.99
Stomach	11 (1.8)	25 (3.6)	.07
Testicular	6 (1)	13 (1.9)	.26
How would you rate your overall health			.68
Very good	48 (7.8)	66 (9.6)	
Good	276 (45.1)	316 (45.8)	
Average	228 (37.3)	243 (35.2)	
Poor	48 (7.8)	45 (6.5)	
Very poor	8 (1.3)	10 (1.4)	
No response	4 (0.7)	10 (1.4)	

a Social nature service that facilitates daily life activities of dependents using technology.

The mean age of patients was 57.8 (SD 13.5) years in the prepandemic survey (2017 study group) study and 58.7 (SD 12.8) years in the postpandemic survey (2023 study group) study. The distribution by age groups did not show statistically significant differences (*P*=.09). Of all the participants in the postpandemic survey (2023 study group) study, 55.4% (382/690) were women, 82.8% (n=571) lived with other people, and 40% (n=276) had a university education. The most common tumor types were breast cancer (n=129, 18.7%), colorectal cancer (n=108, 15.7%), and lung cancer (n=80, 11.6%). Patients’ self-perception of health status was comparable across both periods (*P*=.68), with 55.4% (n=382) rating their health as “good” or “very good.”

Significant differences were observed in the distribution of some tumor types between periods, particularly hematological malignancies (2017: 3/612, 0.5% vs 2023: 54/690, 7.8%; *P*<.001), prostate cancer (2017: 12/612, 2% vs 2023: 51/690, 7.4%; *P*<.001), and sarcoma (2017: 16/612, 2.6% vs 2023: 38/690, 5.5%; *P*=.01). These differences were considered when interpreting temporal changes in ICT use.

### Use of ICTs to Search for Health Information

**Figure 1.**
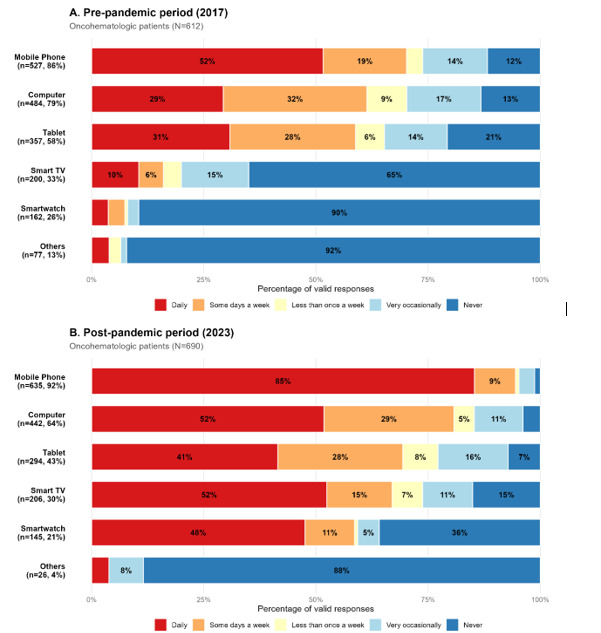
Frequency of information and communication technology use to search for information among patients with cancer (before and after the pandemic).

Figure 1 illustrates the frequency with which patients with cancer used different devices to search for information on the internet. Figure 1 is based on valid responses to the frequency question for each device and is not limited to smartphone users. Table 2 summarizes the proportion of participants who reported using each type of device to search for information on the internet, as well as internet use habits, familiarity with technological terms, and information-seeking behaviors between the 2 study periods.

**Table 2.** Frequency of internet access using digital technologies among patients with cancer.

Question	Prepandemic survey (2017 study group; n=612), n (%)	Postpandemic survey (2023 study group; n=690), n (%)	*P* value
Which of the following terms have you heard before?			
Mobile app	413 (67.5)	525 (76.1)	<.001
Smartphone	404 (66)	494 (71.6)	.03
Information and communication technologies	179 (29.2)	221 (32)	.30
I have not heard any of these terms before	142 (23.2)	102 (14.8)	<.001
eHealth	88 (14.4)	114 (16.5)	.32
Mobile health or mHealth^a^	66 (10.8)	111 (16.1)	.007
Wearable	51 (8.3)	74 (10.7)	.17
Which devices do you use to look for information on the internet?			
Mobile phone	445 (72.7)	624 (90.4)	<.001
Desktop and/or laptop computer	416 (68)	423 (61.3)	.01
Tablet	274 (44.8)	270 (39.1)	.04
Television with internet (Smart TV)	58 (9.5)	161 (23.3)	<.001
Smartwatch	15 (2.5)	89 (12.9)	<.001
Others	3 (0.5)	3 (0.4)	.99
I do not look for information on the internet	84 (13.7)	50 (7.2)	<.001
Are you interested in staying informed about health-related matters?			<.001
Yes	532 (86.9)	564 (81.7)	
No	60 (9.8)	113 (16.4)	
No response	20 (3.3)	13 (1.9)	
Where do you search for information on health?			
Health professionals	462 (75.5)	501 (72.6)	.26
Internet	375 (61.3)	407 (59)	.43
People close to me (friends, relatives, and workmates)	160 (26.1)	122 (17.7)	<.001
Newspapers, magazines, and pamphlets	151 (24.7)	71 (10.3)	<.001
Mobile apps	62 (10.1)	69 (10)	.99
Others	23 (3.8)	28 (4.1)	.89
If you use the internet to search for health information, which types of website do you use?			
Google	436 (71.2)	471 (68.3)	.27
I do not search for medical information on the internet	129 (21.1)	155 (22.5)	.59
Medical societies	124 (20.3)	90 (13)	<.001
Patient associations	123 (20.1)	87 (12.6)	<.001
YouTube	68 (11.1)	84 (12.2)	.61
Facebook	45 (7.4)	29 (4.2)	.02
Blogs	43 (7)	26 (3.8)	.01
Others	11 (1.8)	15 (2.2)	.77
Twitter	10 (1.6)	10 (1.4)	.96
Social networks	32 (5.2)	44 (6.4)	.44
For what purposes do you search for health information?			
Disease prevention, healthy lifestyles, and health care	301 (49.2)	314 (45.5)	.20
To find information about the treatment prescribed by my physician	289 (47.2)	299 (43.3)	.18
To find symptoms and learn about potential diseases	145 (23.7)	194 (28.1)	.08
To find information about medical centers or health professionals	139 (22.7)	107 (15.5)	.001
To find information about alternative or complementary medicines	107 (17.5)	72 (10.4)	<.001
To connect with other people with health problems	71 (11.6)	57 (8.3)	.05
Others	69 (11.3)	69 (10)	.51
Is it easy to understand the health information you find on the internet?			<.001
Usually	269 (44)	231 (33.5)	
Sometimes	148 (24.2)	168 (24.3)	
Always	66 (10.8)	144 (20.9)	
Never	51 (8.3)	67 (9.7)	
No response	78 (12.7)	80 (11.6)	
Do you trust the health information you find on the internet?			.03
Yes	84 (13.7)	64 (9.3)	
No	129 (21.1)	163 (23.6)	
Depends on the website	336 (54.9)	396 (57.4)	
No response	63 (10.3)	67 (9.7)	
Do you look up information before going to your doctor’s appointment?			.99
No	133 (21.7)	147 (21.3)	
Yes	451 (73.7)	500 (72.5)	
No response	28 (4.6)	43 (6.2)	
Do you look up information *after *going to your doctor’s appointment?			.71
Yes	143 (23.4)	156 (22.6)	
Only if I still have doubts	168 (27.5)	179 (25.9)	
No	270 (44.1)	319 (46.2)	
No response	31 (5.1)	36 (5.2)	

a mHealth: mobile health.

Regarding the frequency of use, a shift toward more frequent internet use was observed across several devices in the postpandemic period (Figure 1B) compared with 2017 (Figure 1A). Mobile phone use became the dominant pattern, with daily use for information seeking rising from 52% (318/612) to 85% (586/690) among valid responses. Smart TVs and smartwatches also showed increased frequency of use among respondents who provided a valid answer for each device, although the overall proportion of patients who reported using smartwatches to search for information remained lower, as shown in Table 2.

A significant increase was observed in mobile phone use for searching information, which rose from 72.7% (445/612) to 90.4% (624/690; *P*<.001). There was also a notable increase in the use of smart TVs, from 9.5% (58/612) to 23.3% (161/690; *P*<.001), and smartwatches, from 2.5% (15/612) to 12.9% (89/690; *P*<.001). In contrast, the use of desktop and laptop computers decreased slightly, from 68% (416/612) to 61.3% (423/690; *P*=.01), as did the use of tablets, from 44.8% (274/612) to 39.1% (270/690; *P*=.04).

Familiarity with technological terms also improved. Knowledge of the term “mobile app” increased from 67.5% (413/612) to 76.1% (525/690; *P*<.001), as did knowledge of “smartphone,” which rose from 66% (404/612) to 71.6% (494/690; *P*=.03). The proportion of patients unfamiliar with any of these terms decreased considerably, from 23.2% (142/612) to 14.8% (102/690; *P*<.001).

The proportion of patients interested in staying informed about health-related matters decreased from 86.9% (532/612) to 81.7% (564/690; *P*<.001). Regarding the main sources of health information, health care professionals remained the primary source (462/612, 75.5% vs 501/690, 72.6%; *P*=.26), followed by the internet (375/612, 61.3% vs 407/690, 59%; *P*=.43). Google continued to be the most frequently used tool for searching medical information (436/612, 71.2% vs 471/690, 68.3%; *P*=.27). However, significant decreases were observed in the use of medical societies (124/612, 20.3% vs 90/690, 13%; *P*<.001), patient associations (123/612, 20.1% vs 87/690, 12.6%; *P*<.001), Facebook (45/612, 7.4% vs 29/690, 4.2%; *P*=.02), and blogs (43/612, 7% vs 26/690, 3.8%; *P*=.01).

The main reasons patients searched for health information were disease prevention and healthy lifestyles (301/612, 49.2% vs 314/690, 45.5%) and understanding the treatment recommended by their physician (289/612, 47.2% vs 299/690, 43.3%). No significant differences were found in searching for information about disease or treatment before (*P*=.99) or after (*P*=.71) medical appointments.

In terms of trust in information found on the internet, the distribution of responses showed significant differences (*P*=.03), although the proportion of patients expressing explicit distrust remained similar (129/612, 21.1% vs 163/690, 23.6%). Most respondents indicated that their trust depended on the website (336/612, 54.9% vs 396/690, 57.4%). The remaining responses regarding the frequency of ICT use for seeking health information are shown in Figure 1.

### Preferences for the Use of Health Apps

Table 3 presents the preferences for the use of health apps among patients with cancer before and after the study period.

**Table 3.** Preferences for the use of health apps.

Question	Prepandemic survey (2017 study group; n=612), n (%)	Postpandemic survey (2023 study group; n=690), n (%)	*P* value
Is your mobile phone a smartphone?			<.001
No	93 (15.2)	41 (5.9)	
Yes	505 (82.5)	628 (91)	
No response	14 (2.3)	21 (3)	
Do you have health-related apps installed?			<.001
No	478 (78.1)	308 (44.6)	
Yes	124 (20.3)	372 (53.9)	
No response	10 (1.6)	10 (1.4)	
What do you use your mobile phone for?			
Normal phone use (calls, messages, etc)	591 (96.6)	661 (95.8)	.56
To access the internet	347 (56.7)	449 (65.1)	.002
Schedule planner and alarms	305 (49.8)	384 (55.7)	.04
To use apps	262 (42.8)	374 (54.2)	<.001
Social networks	233 (38.1)	346 (50.1)	<.001
How would you like to communicate with your health professional?			
Telephone	470 (76.8)	483 (70)	.007
Email	270 (44.1)	179 (25.9)	<.001
Mobile apps	250 (40.8)	134 (19.4)	<.001
Videoconference	116 (19)	221 (32)	<.001
Website	51 (8.3)	10 (1.4)	<.001
Blogs	20 (3.3)	5 (0.7)	.002
Social networks	18 (2.9)	13 (1.9)	.29
Would you use an app if your health professional recommended it?			.99
Yes	498 (81.4)	537 (77.8)	
No	82 (13.4)	88 (12.8)	
No response	32 (5.2)	65 (9.4)	

A significant increase was observed in the use of smartphones, rising from 82.5% (505/612) to 91% (628/690; *P*<.001), while the proportion of patients not using a smartphone decreased markedly from 15.2% (93/612) to 5.9% (41/690). In addition, the number of patients with health-related apps installed increased from 20.3% (124/612) to 53.9% (372/690), with a clear reduction in those who did not have such apps (478/612, 78.1% to 308/690, 44.6%; *P*<.001). The questionnaire captured whether patients had health-related apps installed, but it did not collect information on app categories, frequency of use, or retention over time.

Regarding mobile phone use, most patients continued to use it for basic functions such as calls, messages, or photos, with no significant changes (591/612, 96.6% vs 661/690, 95.8%; *P*=.56). However, internet access via mobile phones increased significantly, from 56.7% (347/612) to 65.1% (449/690; *P*=.002). The use of mobile phones for managing schedules and alarms also rose (305/612, 49.8% to 55.7%; *P*=.04), as did the use of apps (262/612, 42.8% to 374/690, 54.2%; *P*<.001) and access to social networks (233/612, 38.1% to 50.1%; *P*<.001).

In terms of communication preferences with health care professionals, the telephone remained the preferred option, although its use declined from 76.8% (470/612) to 70% (483/690; *P*=.007). The preference for email also decreased considerably, from 44.1% (270/612) to 25.9% (179/690; *P*<.001), as did the preference for mobile apps, which dropped from 40.8% (250/612) to 19.4% (134/690; *P*<.001). In contrast, the preference for videoconferencing as a communication channel increased significantly from 19% (116/612) to 32% (221/690; *P*<.001).

Finally, regarding the hypothetical use of an app recommended by a health care professional, most patients indicated they would use it, although the proportion decreased slightly from 81.4% (498/612) to 77.8% (537/690; *P*=.99). The proportion of patients who affirmed they would not use it remained low and stable (82/612, 13.4% vs 88/690, 12.8%).

### Impact of COVID-19 on ICT Use

Participants were asked about the impact of the COVID-19 pandemic on their access to health care and use of technology. Regarding the accessibility of medical care through ICTs during this period, opinions were divided. While 33.5% (231/690) of patients considered that care was “more accessible,” a slightly higher proportion (n=232, 33.6%) responded “I don’t know.” Only 4.6% (n=32) felt that access was “less accessible,” and 24.8% (n=171) considered it “equally accessible.”

Regarding the specific use of mobile apps during the pandemic, the majority of patients (290/690, 42%) reported that their use remained “the same.” However, 27.1% (n=187) reported “greater use” of apps. A quarter of the respondents (n=174, 25.2%) stated that they do not use apps at all.

Finally, patients’ views on telemedicine compared to traditional consultations showed a tendency toward a negative perception. The most frequent response was that telemedicine is “worse” (261/690, 37.8%), followed by those who viewed it as “positive” (n=228, 33%) or were “indifferent” (n=153, 22.2%). The complete data regarding the impact of COVID-19 are shown in Table 4.

**Table 4.** Impact of COVID-19 on the use of new information and communication technologies.

Question	Postpandemic survey (2023 study group; n=690), n (%)
As a result of the COVID-19 pandemic, do you think access to online health content and apps is	
Equally accessible	171 (24.8)
I do not know	232 (33.6)
Less accessible	32 (4.6)
More accessible	231 (33.5)
No response	24 (3.5)
Since the COVID-19 pandemic, has your use of apps for health-related queries changed?	
Greater use	187 (27.1)
I do not use apps	174 (25.2)
Less use	12 (1.7)
No response	27 (3.9)
The same	290 (42)
How do you view the promotion of telemedicine and applications as a result of COVID-19?	
Indifferent	153 (22.2)
No response	24 (3.5)
Other	24 (3.5)
Positive	228 (33)
Worse	261 (37.8)

## Discussion

### Principal Findings

The main findings of this study are that patients with cancer reported greater adoption of digital technologies in 2023 than in 2017, particularly in smartphone use and installation of health-related apps, but this increase did not translate into full trust in online information or into a clear preference for apps as a communication channel with health care professionals. Smartphone penetration reached 91% (628/690) in 2023 compared with 82.5% (505/612) in 2017, and the use of health-related apps increased from 20.3% (124/612) to 53.9% (372/690) [6]. This shift is consistent with the global expansion of the health app market, which currently includes approximately 337,000 tools, and with the impact of the pandemic on the demand for mHealth services [2,5-8].

### Comparison With Previous Studies

In line with these findings, recent studies in immune-mediated diseases also report broad acceptance of ICTs for chronic disease management, with 42.8% of patients already using specific apps and 90% willing to do so if recommended by their health care professional [12]. Patient-driven reviews in cancer have also emphasized that digital health portals and apps should be designed around patients’ needs and preferences [13,14]. Our results are consistent with this trend, showing that most patients with cancer (537/690, 77.8%) would use a health app if recommended by their health care professional. Familiarity with digital concepts also improved: the proportion of patients unfamiliar with terms such as “mobile app” or “smartphone” decreased substantially (102/690, 14.8% in 2023 vs 142/612, 23.2% in 2017), suggesting a general improvement in digital literacy among this population [15,16].

However, the increase in mobile device use has not translated into greater confidence in online information. In both periods analyzed, only a minority expressed full trust in the content they found online, and in 2023, only 9.3% (64/690) reported full trust. Most patients stated that trust was contingent on source credibility, suggesting that patients do not reject online information itself but need clear signals about reliability, institutional endorsement, and professional validation. This phenomenon has also been reported in recent studies, where only 9% of patients expressed trust in digital health content [17].

A particularly relevant finding is the evolution of communication preferences with health care professionals. Although greater uptake of asynchronous channels might be expected, our data show a significant decline in the preference for email (179/690, 25.9% in 2023 vs 270/612, 44.1% in 2017) and mobile apps (134/690, 19.4% in 2023 vs 250/612, 40.8% in 2017). By contrast, videoconferencing increased significantly from 19% (116/612) to 32% (221/690; *P*<.001), while telephone calls remained the preferred option (483/690, 70% in 2023 vs 470/612, 76.8% in 2017; *P*=.007). This increase may be related to greater familiarity with video consultations after the pandemic and the ability of videoconferencing to preserve visual contact and nonverbal communication, which are important components of the therapeutic relationship [18,19]. In Spain, although 72.5% of users access online medical appointments, only 17.8% use digital channels to communicate with health care professionals, highlighting a mismatch between technological supply and user preferences [20].

The gap between willingness to use recommended apps (537/690, 77.8%) and preference for apps as a communication channel (134/690, 19.4%) is clinically relevant. It suggests that patients may accept an app when it has a clear purpose, professional recommendation, and perceived usefulness but may not want apps to replace established communication channels. This interpretation is also consistent with the decline observed in interest in staying informed about health-related matters (532/612, 86.9% to 564/690, 81.7%) and in the use of patient associations and medical societies as information sources. Rather than indicating a lower need for information, this may reflect a more selective approach to information seeking, greater reliance on health care professionals, or difficulties identifying trustworthy digital sources.

The differences in cohort composition between periods should also be considered. The postpandemic survey (2023 study group) cohort included a higher proportion of patients with hematological malignancies, prostate cancer, and sarcoma than the prepandemic survey (2017 study group) cohort. Tumor type, treatment modality, route of administration, visit frequency, age profile, and symptom burden may influence technology use, preferences for remote communication, and perceived usefulness of apps. Therefore, changes over time should be interpreted as global trends in our surveyed population rather than as effects exclusively attributable to the COVID-19 pandemic.

Despite advances in digital adoption, structural barriers persist. Difficulties in using digital tools remain relevant, especially among older adults and people with limited digital skills. Data from Europe show persistent gaps in digital competencies among older populations [21], and implementation studies emphasize the importance of addressing nonuse, abandonment, and long-term retention of digital health technologies [22]. In our study, the finding that 33.6% (232/690) of patients answered “I don’t know” when asked whether online health content and apps were more accessible after COVID-19 suggests that improved availability does not necessarily translate into perceived accessibility or usefulness for all patients.

### Limitations

This study has several limitations. Its cross-sectional design may limit causal inferences, and the temporal gap between the prepandemic survey (2017 study group) and postpandemic survey (2023 study group) means that factors other than the COVID-19 pandemic may have contributed to the observed changes. In addition, the cohort composition differed between periods, with a higher proportion of patients with hematological malignancies, prostate cancer, and sarcoma in the postpandemic survey (2023 study group) cohort; these differences may have influenced ICT use patterns and communication preferences. The use of self-administered, paper-based questionnaires may have introduced selection or comprehension biases and may have underrepresented patients with greater digital exclusion, potentially overestimating ICT adoption. The questionnaire was based on a previous survey and was reviewed for comprehension by 4 patients, but it was not a standardized or psychometrically validated instrument. Missing responses were reported as “no response” and were not imputed. Finally, the questionnaire captured whether health apps were installed, but it did not characterize app type, frequency of use, long-term adoption, or retention. As the study was conducted in a single center, the findings may not be generalizable to other populations or health care settings. However, the large sample size enhances the precision of the estimates and supports the robustness of the findings across similar settings.

### Conclusions

This study reflects a positive trend in the adoption of digital technologies by patients with cancer in recent years, particularly after the COVID-19 pandemic. Increased familiarity with digital concepts and more frequent use of smartphones as health tools were observed. However, barriers remain, including concerns about trust in online information, disparities in digital skills, and a potential mismatch between available technological solutions and users’ real preferences. These results suggest that digital health strategies in oncology should prioritize clinician-curated information sources, patient co-design, clear recommendations of reliable apps, simple and secure communication channels, and targeted support for patients with lower digital literacy, while preserving nondigital alternatives when needed.

## Supplementary material

10.2196/90339 Multimedia Appendix 1 Survey: use of information and communication technologies among patients with cancer.

## Abbreviations

ICT: information and communication technology
mHealth: mobile health
