# Supplementary material for: Use of Information and Communication Technologies in Patients With Cancer Receiving Antineoplastic or Supportive Therapy: Comparative Cross-Sectional Survey
Source: J Med Internet Res. 2026 Aug 6;28:e90339. doi: 10.2196/90339 (PMC13447052; doi:10.2196/90339)
Supplement: Supplementary file 1 [file jmir-v28-e90339-s001.docx]

**SURVEY:**

**Use of information and communication technologies in oncology patients**

This survey aims to understand the extent to which patients with cancer use new information and communication technologies. Throughout the survey, we will ask about different aspects of using mobile technology (internet and mobile applications) related to health.

Your responses are confidential and anonymous. The information collected will help us design improvements that enhance care for patients with cancer.

Please read each question carefully and mark with an “X” the option(s) that apply to you. You may select more than one option when appropriate.

**Section A. Sociodemographic characteristics**

**1. Date of birth:**

**2. Sex**

☐ Male

☐ Female

☐ Other

**3. Do you live alone?**

☐ Yes

☐ No

**4. Education level**

☐ No formal education or incomplete primary education

☐ Primary education

☐ Secondary education

☐ University education

**5. Do you have any degree of functional dependence?**

☐ No

☐ I need help once a day

☐ I need help several times a day

☐ I need help all the time

**6. Do you use any home telecare service?**

☐ Yes

☐ No

**7. What type of tumor do you have?**

☐ Acute leukemia

☐ Bladder

☐ Breast

☐ Cervix

☐ Chronic lymphocytic leukemia

☐ Chronic myeloid leukemia

☐ Colorectal

☐ Esophagus

☐ Head and neck Stomach

☐ Kidney

☐ Lung

☐ Lymphoma

☐ Multiple myeloma

☐ Ovary

☐ Pancreas

☐ Prostate

☐ Sarcoma

☐ Skin – Melanoma

☐ Testicle

☐ Other

**8. How would you rate your overall health?**

☐ Very good

☐ Good

☐ Fair

☐ Poor

☐ Very poor

**Section B. Use of information and communication technologies (ICTs) to search for health information**

**9. Which of the following terms have you heard of?** *(You may select multiple options)*

☐ ICT (Information and Communication Technologies)

☐ eHealth

☐ mHealth (mobile health)

☐ Smartphone

☐ App (mobile application)

☐ Wearable

☐ I have not heard of any of these terms

**10. Do you use devices to search for information on the internet?** *(You may select multiple options)*

☐ Mobile phone

☐ Desktop and/or laptop computer

☐ Tablet

☐ Internet-connected TV (Smart TV)

☐ Smartwatch and/or fitness band (wearable)

☐ Other(s)

☐ I do not use any device

**11. How often do you use them?**

|  | Daily | Some days a week | Less tan once a week | Very occasionally | Never |
| --- | --- | --- | --- | --- | --- |
| Teléfono móvil Mobile phone |  |  |  |  |  |
| Desktop and/or laptop computer |  |  |  |  |  |
| Tablet |  |  |  |  |  |
| SmartTV) |  |  |  |  |  |
| Smartwatch or fitness band (wearable) |  |  |  |  |  |
| Other |  |  |  |  |  |

**12. Are you interested in staying informed about health topics?**

☐ Yes

☐ No

**13. Where do you look for health information?** *(You may select multiple options)*

☐ Healthcare professionals

☐ Close contacts (family, friends, coworkers)

☐ Newspapers, magazines, brochures

☐ Internet

☐ Mobile applications (apps)

☐ Other(s)

**14. If you use the internet to search for health information, which types of sites do you use?** *(You may select multiple options)*

☐ Google

☐ Blogs

☐ YouTube

☐ Social networks – Twitter/X

☐ Social networks – Facebook

☐ Social networks – Other

☐ Patient associations

☐ Medical societies

☐ Other(s)

☐ I do not search for medical information online

**15. For what purpose do you search for health information?** *(You may select multiple options)*

☐ Disease prevention, healthy lifestyles, self-care

☐ Looking up symptoms to learn about possible diseases

☐ Learning about the treatment prescribed by my doctor

☐ Learning about complementary or alternative medicines

☐ Finding information about medical centers or healthcare professionals

☐ Connecting with other people with health problems similar to mine

☐ Other(s)

**16. Is the health information you find online easy to understand?**

☐ Always

☐ Often

☐ Sometimes

☐ Never

**17. Do you trust the health information you find online?**

☐ Yes

☐ No

☐ It depends on the website

**18. Do you look up information online about your disease and/or treatment before your medical appointment?**

☐ Yes

☐ No

**19. Do you look up information online about your disease and/or treatment after your medical appointment?**

☐ Yes

☐ Only if I still have questions

☐ No

**Section C. Preferences for the use of health apps**

**20. Is your mobile phone a smartphone? (touchscreen, allows internet access, email, etc.)**

☐ Yes

☐ No

**21. What do you use your mobile phone for?** *(You may select multiple options)*

☐ Phone use (calls, messages, photos/videos, etc.)

☐ Social media

☐ Calendar and alarms

☐ Browsing the internet

☐ Using applications (apps)

**22. Do you have health-related mobile apps?**

☐ Yes

☐ No

**23. What do you use health-related apps for?** *(You may select multiple options)*

☐ Preventing diseases, addressing health problems, and improving my lifestyle

☐ Obtaining information about my disease and its treatments

☐ Managing appointments with my clinic, hospital, or healthcare professional

☐ Recording and tracking my medication

☐ Recording and tracking my symptoms

☐ Contacting healthcare professionals

☐ Remote monitoring by healthcare professionals

☐ Emotional support

☐ Other(s)

**24. What would you like a health-related app to help you with?** *(You may select multiple options)*

☐ Preventing diseases, addressing health problems, and improving my lifestyle

☐ Obtaining information about my disease and its treatments

☐ Managing appointments with my clinic, hospital, or healthcare professional

☐ Recording and tracking my medication

☐ Recording and tracking my symptoms

☐ Contacting healthcare professionals

☐ Remote monitoring by healthcare professionals

☐ Emotional support

☐ Other(s)

**25. How would you like to communicate with your healthcare professional?** *(You may select multiple options)*

☐ Email

☐ Telephone

☐ Mobile application (app)

☐ Social media

☐ Blog

☐ Website

☐ Videoconference

**26. How did you find the health-related apps you use?** *(You may select multiple options)*

☐ Recommendation from a family member and/or friend

☐ Prescription and/or recommendation from a healthcare professional

☐ On my own initiative

**27. Would you use an app if your healthcare professional recommended it?**

☐ No

☐ Probably

☐ Yes

☐ Yes, even if I had to pay to download it

**Section D.** **Impact of COVID-19 on new information and communication technologies use**

**28. Do you think that, as a result of the COVID‑19 pandemic, access to online health content and other technologies such as apps is:**

☐ Equally accessible

☐ More accessible

☐ Less accessible

☐ I don’t know

**29. Since the COVID‑19 pandemic, your use of apps for health-related queries is:**

☐ Greater use

☐ Less use

☐ The same

☐ I do not use apps

**30. How do you view the promotion of telemedicine and the use of applications as a result of COVID‑19?**

☐ It is positive; I prefer to get informed and consult because it is more direct and faster

☐ Indifferent; I think both approaches are compatible

☐ It is worse; it undermines in-person contact with healthcare professionals

☐ Other

**Please add any comments on the topic below:**

_________________________________________________________________________________________________________

_________________________________________________________________________________________________________

_________________________________________________________________________________________________________

_________________________________________________________________________________________________________

_________________________________________________________________________________________________________

_________________________________________________________________________________________________________
